# Update of Brazilian Guidelines for Treatment and Assessment of Chronic Kidney Disease – Mineral and Bone Disorders

**DOI:** 10.1590/2175-8239-JBN-2021-S101

**Published:** 2021-12-03

**Authors:** Aluízio Barbosa Carvalho, Fellype Carvalho Barreto

**Affiliations:** 1 Universidade Federal de São Paulo, Faculty of Medicine, Nephrology Discipline, São Paulo, SP, Brazil.; 2 Universidade Federal do Paraná, Faculty of Medicine, Department of Internal Medicine, Curitiba, PR, Brazil.

The previous version of the guideline dates from 2011, a time when the applicability of bone densitometry in the assessment of patients with chronic kidney disease was questionable; the treatment of osteoporosis was almost a taboo; we were still in the early stages of incorporating paricalcitol and cinacalcet into the therapeutic arsenal available for treatment of secondary hyperparathyroidism in our country; and the CKD-MBD department was still considered a committee. The publication of the updated Kidney Disease: Improving Global Outcomes (KDIGO) CKD-MBD guidelines, along with the publication of new clinical studies related to diagnosis and treatment of CKD-MBD^1^, naturally led to the need for revision and updating of Brazilian Guidelines by the current Brazilian Society of Nephrology (BSN) CKD-MBD Department. 

In August 2019, at the BSN headquarters, the meeting that commenced the preparation of the guidelines update was held. We could not imagine that, a few months later, we would be facing the COVID-19 pandemic, which made that first meeting the only one of the guidelines group and the last one where we could be together in person. Given the new and growing professional and personal demands that have abruptly become part of our daily lives, redoubled efforts were required from all of us so that the guidelines, which inevitably had become secondary, would not leave our focus. Solidarity and friendship among guideline developers, unrestricted support from the Brazilian Society of Nephrology, and commitment to delivering the new guidelines to fellow nephrologists gave us the necessary strength to complete them by mid 2021. 

The current version is structured into three sections. The first one, focused on the CKD-MBD diagnosis (chapters 1 to 3); the second, on the treatment (chapters 4 to 10); and the third one, which we named miscellaneous (chapters 11 to 13), for comprising different areas, such as parathyroidectomy, kidney transplantation and pediatric nephrology. In the updated version, issues that have gained great relevance in recent years and that had not been addressed in the previous version, such as calciphylaxis, osteoporosis, and the use of bone densitometry in patients with chronic kidney disease, have been incorporated. The multidisciplinary character of the guidelines was enhanced by the collaboration of colleagues from head and neck surgery, along with the traditional involvement of nutritionists and nephropediatricians. Similarly to previous versions of the Guidelines (2008 and 2011)^2^ ^,^ ^3^, the term "Evidence" was assigned whenever it was based on evidence published in literature, regardless of its degree. Otherwise, the term "Opinion" was used, resulting from opinions within the consulted Guidelines, often adapted to the personal experience of this forum.
